# Symbionts on the Brain: How *Wolbachia* Is Strictly Corralled in Some Neotropical *Drosophila* spp.

**DOI:** 10.1128/mbio.01182-22

**Published:** 2022-07-14

**Authors:** Denis Voronin, Benjamin L. Makepeace

**Affiliations:** a Systems Genomics Section, Laboratory of Parasitic Diseases, Division of Intramural Research, NIAID, NIH, Bethesda, Maryland, USA; b Institute of Infection, Ecological & Veterinary Sciences, University of Liverpool, Liverpool, United Kingdom

**Keywords:** autophagy, endoplasmic reticulum, neuroblast, symbiosis

## Abstract

*Wolbachia* is a heritable alphaproteobacterial symbiont of arthropods and nematodes, famous for its repertoire of host manipulations, including cytoplasmic incompatibility. To be vertically transmitted, *Wolbachia* must efficiently colonize the female germ line, although somatic tissues outside the gonads are also infected. In *Drosophila* spp., *Wolbachia* is usually distributed systemically in multiple regions of the adult fly, but in some neotropical hosts, *Wolbachia*’s only somatic niches are cerebral bacteriocyte-like structures and the ovarian follicle cells. In their recent article, Strunov and colleagues (A. Strunov, K. Schmidt, M. Kapun, and W. J. Miller. mBio 13:e03863-21, 2022, https://doi.org/10.1128/mbio.03863-21) compared the development of *Drosophila* spp. with systemic or restricted infections and demonstrated that the restricted pattern is determined in early embryogenesis by an apparently novel autophagic process, involving intimate interactions of *Wolbachia* with the endoplasmic reticulum. This work has implications not only for the evolution of neotropical *Drosophila* spp. but also for our understanding of how *Wolbachia* infections are controlled in other native or artificial hosts.

## COMMENTARY

*Wolbachia* is a heritable alphaproteobacterial symbiont of arthropods and nematodes, with a remarkably broad host range, global distribution, and repertoire of host manipulations. In arthropods, it is most famous for two phenotypic effects: cytoplasmic incompatibility (CI) and pathogen protection. In CI, crosses between infected males and uninfected females, or females harboring an incompatible *Wolbachia* strain, are rendered inviable due to modification of sperm by symbiont-derived protein effectors. In females infected with a compatible strain, embryonic development is “rescued” by an effector produced by *Wolbachia* in the ovaries (reviewed in reference 1). Thus, CI confers a benefit to compatibly infected females, resulting in successful spread of *Wolbachia* through the population. Pathogen protection is a second major *Wolbachia* phenotype that can also engender enhanced fitness in infected arthropods and is beginning to have a major translational impact through mass release of transinfected mosquitoes, which are incompetent for arbovirus transmission (reviewed in reference 2).

*Wolbachia*’s reproductive manipulations such as CI are dependent on infection of the gonads of both sexes, and especially the female germ line, as a prerequisite for vertical transmission. While *Wolbachia* strain *w*Mel directly targets the germ line precursor cells of Drosophila melanogaster during embryogenesis (3), this is not the only mechanism by which the female germ line is colonized; indeed, *Wolbachia* strains in other Old World *Drosophila* species do not target germplasm during embryogenesis (4). Rather, they show a remarkable tropism for the somatic stem cell niche. From this location, *Wolbachia* can effectively colonize the germ line during oogenesis. Moreover, it can even target the somatic cell niche if artificially introduced into adult female flies (5).

In most arthropod hosts, *Wolbachia* is not confined to a bacteriocyte and can be detected in several somatic tissues outside the gonads, such as the central nervous system (CNS), hemocytes, and muscles. The evolutionary significance of these systemic infections remains enigmatic, although there is evidence of pathogen protection in wild populations of *Drosophila* (6), and a wide symbiont tissue distribution may facilitate this. Systemic infections can also affect host behavior, a striking example of which is found in the neotropical Drosophila paulistorum complex. In addition to hybrid male sterility and bidirectional CI, strong premating behavioral barriers between semispecies in this complex are induced by obligate *Wolbachia* symbionts (7). Consequently, upon antibiotic treatment to reduce *Wolbachia* titer, female mate choice for males of sympatric semispecies becomes random rather than assortative (7). Thus, the radiation of the *D. paulistorum* complex in *statu nascendi* constitutes a paradigm of “infectious speciation,” with *Wolbachia* driving reproductive isolation.

Unlike Old World *Drosophila* spp. such as D. melanogaster, *D. paulistorum* and the related Drosophila willistoni exhibit highly restricted *Wolbachia* distributions. In developing embryos, *Wolbachia* are largely confined to the primordial germ cells, whereas somatic infections in adult flies are circumscribed within bacteriocyte-like structures located in the brain (8). Here, it is hypothesized that *Wolbachia* acts as a “puppet-master,” controlling the sexual behavior of its host to avoid conflict with incompatible *Wolbachia* strains in the species complex.

In an elegant new study, Strunov and colleagues (9) uncover the cellular basis of restricted *Wolbachia* infections in three neotropical species (*D. paulistorum*, *D. willistoni*, and Drosophila sturtevanti) and demonstrate that two other neotropical species (Drosophila tropicalis and Drosophila septentriosaltans) harbor systemic infections akin to those of D. melanogaster and other Old World species. The authors examined infection patterns in the CNS of third-stage larvae and determined that while *Wolbachia* were present in glial cells and neurons in all species examined, in hosts with restricted infections, type I neuroblasts were infected, whereas in systemic infections, type I and II neuroblasts were colonized, permitting a wider distribution in the adult brain.

Clear differences between systemic and restricted infections were also demonstrated in the female gonad. Systemic infections were more widely distributed in the somatic regions of the ovary, whereas only the follicle cells were targeted in restricted infections. Furthermore, *Wolbachia* infections were more focal in follicle cells and attained higher densities in the germ line (nurse cells and oocytes) in the “restricted” hosts compared with “systemic” ones. As with the neural infections, these distinct patterns between restricted and systemic host species were already apparent during larval development.

The key question is how does *Wolbachia* become confined to strictly prescribed niches? To answer this, Strunov and colleagues (9) followed *Wolbachia* distribution during embryogenesis in D. melanogaster and the five neotropical *Drosophila* spp. In early embryos, *Wolbachia* distribution was very similar between all six host species. However, as gastrulation commenced in mid embryogenesis, *Wolbachia* densities declined in restricted hosts. By late embryogenesis, the systemic and restricted patterns had diverged dramatically, with restricted infections becoming confined to primordial germ cells, gonad cells, and a few somatic cell clusters. Indeed, differences in neuroblast infection observed in third-stage larvae were already apparent by mid- to late embryogenesis. The authors hypothesized that autophagy was responsible for restricted infections, as this process is known to regulate *Wolbachia* density in D. melanogaster gonads (10), as well as in various tissues of *Wolbachia*-infected filarial nematodes (11). Accordingly, an autophagosome-specific marker revealed rings around *Wolbachia* cells in restricted host species only, with a peak in autophagy during early gastrulation. Importantly, no autophagosomes were detected in primordial germ cells, where infection appeared to be tolerated by the host.

Further investigations using transmission electron microscopy of embryos during cellularization and early gastrulation revealed *Wolbachia* in close proximity to rough endoplasmic reticulum (ER) and abnormal symbiont morphology in restrictive but not systemic hosts. Of all host species examined, only *D. willistoni* exhibited clear evidence of symbiont tagging by ubiquitin. In third-stage larvae and adult flies, no evidence for autophagy controlling symbiont location or titer was found, indicating that the restriction of infection is wholly determined prior to larval development. The authors asked next if the different phenotypes were a property of the *Wolbachia* strain or *Drosophila* host. Drosophila simulans was cleared of its native systemic infection with strain *w*Au and transinfected with strain *w*Wil from *D. willistoni.* In the new host, *w*Wil became systemic, and neither control via autophagy during embryogenesis nor ubiquitination of the introduced symbiont were apparent.

**FIG 1:**
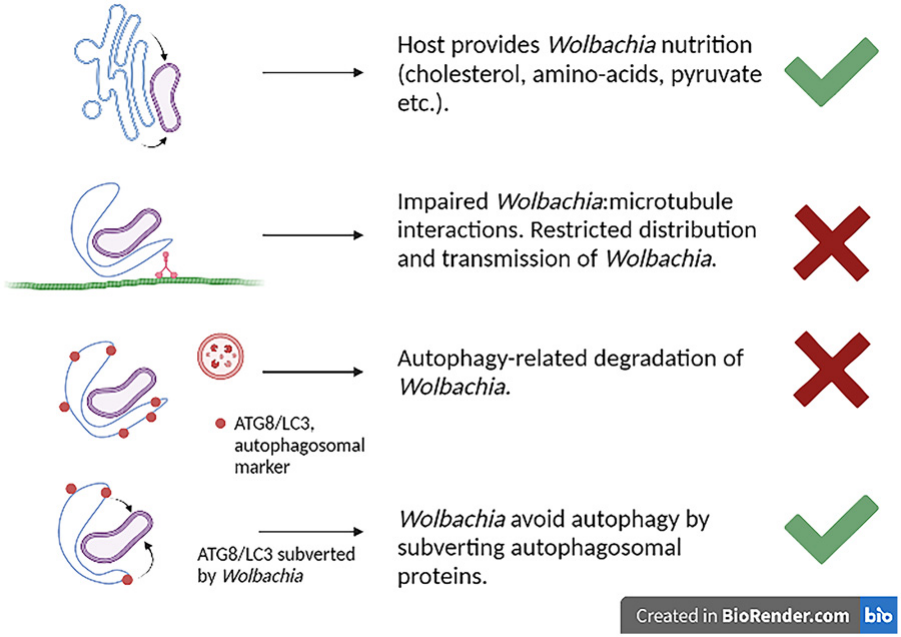
Interactions with the ER determine *Wolbachia* tissue locations in *Drosophila* spp. In cellular locations where the host tolerates *Wolbachia*, the symbiont acquires essential nutrients from the organelle. *Wolbachia* is excluded from most somatic tissues by late embryogenesis (restricted phenotype) or from a narrower range of tissues later in development (systemic phenotype), perhaps due to breakdown of *Wolbachia*-microtubule interactions during mitosis. In the restricted phenotype, the ER initiates autophagy and degradation of *Wolbachia* in precursor cells of most somatic tissues. In other somatic and germ line cells where *Wolbachia* is tolerated in the adult fly, the symbiont subverts autophagy and avoids the ER stress response.

It is important to emphasize that systemic and restricted *Wolbachia* infections are not strictly dichotomous; the former lead to neither random nor uniform somatic tissue distributions in the adult fly, although infection is substantially more dispersed than in the restricted phenotype. While *Wolbachia* is distributed symmetrically and is dependent on association with microtubules of the centrosome in early D. melanogaster and *D. simulans* embryos, asymmetric segregation predominates in late embryogenesis and larval neurogenesis (12). *Wolbachia* then partitions selectively with the self-renewing apical neuroblast, not the basal small ganglion mother cell, leading to a broad, but not ubiquitous, distribution in the adult fly brain (12). These observations suggest that a process of symbiont containment or elimination occurs at later stages of development in the systemic phenotype. Overall, it appears that *Wolbachia*’s interactions with the ER are a double-edged sword (Fig. 1). Subversion of the ER is clearly tolerated in many somatic tissues of the adult fly, providing the symbiont with nutrients (13). However, in restricted infection, the association with ER membranes might impede *Wolbachia*’s microtubule-dependent movement, and this could be the trigger for selective elimination by autophagy (Fig. 1). In systemic infection, a degree of tissue restriction still occurs in later stages of development through an unknown mechanism, perhaps involving a breakdown in ER-mediated nutrient acquisition by *Wolbachia*.

These findings have a number of important implications for *Wolbachia* research and the wider field of inherited arthropod symbionts. In the context of the *D. paulistorum* complex, this study raises the question of how *Wolbachia* and the host cooperate to maintain high-titer infections in critical sites such as the type II neuroblasts through embryogenesis, leading to formation of cerebral bacteriocyte-like structures. More widely, this work underlines our ignorance about the relative contribution of *Wolbachia* and host genes to symbiont phenotype. Whereas the *Wolbachia* strains infecting most neotropical *Drosophila* spp. are related to *w*Au and cause either systemic or restricted infections, *w*Stv in *D. sturtevanti* is phylogenetically distinct and has a restricted distribution. Although the transinfection experiments in this study strongly support a dominant role for the host, it is known from previous work that *Wolbachia* strain is more important than host background for gonadal tissue tropism in Old World *Drosophila* spp. (4).

It is clear that autophagy is a lynchpin in regulation of tissue tropism and titer in *Wolbachia* infections. However, how *Wolbachia* becomes pathogenic in certain situations while the host retains tight control via autophagy in others remains largely unresolved. For instance, the artificially selected strain *w*MelPop remains pathogenic when transferred between fruit flies and mosquitoes (14), whereas in the case of *w*VulC in isopods, transfer from a native to artificial host causes death of the recipient by uncontrolled autophagy (15). A striking example of host background underpinning pathology occurs in F1 male hybrids of *D. paulistorum* semispecies, in which *Wolbachia* overreplicates in the testes, causing infertility (7). Yet, as the success of *Wolbachia* transinfections into *Aedes* spp. for arbovirus control demonstrates, it is unusual for novel *Wolbachia* infections to be pathogenic.

In conclusion, Strunov and colleagues (9) have revealed key mechanistic insights into the regulation of tissue tropism in neotropical *Drosophila* species. While of central importance in unravelling the nature of the symbiosis in this sublime model of speciation, their research also pushes forward fundamental discoveries in arthropod-symbiont interactions and cell biology, not least for the fast-moving autophagy field.
